# Activation of *T-bet*, *FOXP3*, and *EOMES* in Target Organs From Piglets Infected With the Virulent PRRSV-1 Lena Strain

**DOI:** 10.3389/fimmu.2021.773146

**Published:** 2021-12-09

**Authors:** Inés Ruedas-Torres, Jaime Gómez-Laguna, José María Sánchez-Carvajal, Fernanda Larenas-Muñoz, Inmaculada Barranco, Francisco José Pallarés, Librado Carrasco, Irene Magdalena Rodríguez-Gómez

**Affiliations:** Department of Anatomy and Comparative Pathology and Toxicology, International Agrifood Campus of Excellence (ceiA3), Faculty of Veterinary Medicine, University of Córdoba, Córdoba, Spain

**Keywords:** transcription factors, cytokines, PRRSV-1, virulence, target organs

## Abstract

Transcription factors (TFs) modulate genes involved in cell-type-specific proliferative and migratory properties, metabolic features, and effector functions. Porcine reproductive and respiratory syndrome virus (PRRSV) is one of the most important pathogen agents in the porcine industry; however, TFs have been poorly studied during the course of this disease. Therefore, we aimed to evaluate the expressions of the TFs *T-bet*, *GATA3*, *FOXP3*, and Eomesodermin (*EOMES*) in target organs (the lung, tracheobronchial lymph node, and thymus) and those of different effector cytokines (*IFNG*, *TNFA*, and *IL10*) and the Fas ligand (*FASL*) during the early phase of infection with PRRSV-1 strains of different virulence. Target organs from mock-, virulent Lena-, and low virulent 3249-infected animals humanely euthanized at 1, 3, 6, 8, and 13 days post-infection (dpi) were collected to analyze the PRRSV viral load, histopathological lesions, and relative quantification through reverse transcription quantitative PCR (RT-qPCR) of the TFs and cytokines. Animals belonging to both infected groups, but mainly those infected with the virulent Lena strain, showed upregulation of the TFs *T-bet*, *EOMES*, and *FOXP3*, together with an increase of the cytokine *IFN-γ* in target organs at the end of the study (approximately 2 weeks post-infection). These results are suggestive of a stronger polarization to Th1 cells and regulatory T cells (Tregs), but also CD4^+^ cytotoxic T lymphocytes (CTLs), effector CD8^+^ T cells, and γδT cells in virulent PRRSV-1-infected animals; however, their biological functionality should be the object of further studies.

## 1 Introduction

The correct functioning of the cell-mediated immune system is a major branch in the successful eradication of viruses (1). After antigen presentation, naive CD4^+^ T cells undergo several rounds of division and become polarized into different effector T helper (Th) cell subsets (2). This process is tightly regulated by a set of transcription factors (TFs) that, together with cytokines in the priming environment, contribute to building a strong and guided adaptive immune response ready to defend the host (2, 3). The protective effect of these CD4^+^ T-cell subsets is achieved, on the one hand, by the production of different effector cytokines and, on the other hand, through direct cytolytic activity mediated by proteins such as granzyme B or Fas (2). This effect is also reached by the participation of cytotoxic T cells and natural killer (NK) cells (4, 5).

T-bet (also known as TBX21) is a member of the T-box family TFs whose expression induces Th1 cell differentiation and promotes the production of tumor necrosis factor alpha (TNF-α) and interferon gamma (IFN-γ) (3, 6, 7). The TF GATA-binding protein 3 (or GATA3) acts as a master regulator for the differentiation of Th2 response; however, it is also involved in the earlier stages of hematopoietic and lymphoid cell development in the thymus (8). FOXP3, belonging to the forkhead box family of TFs, plays a role in the differentiation of regulatory T cells (Tregs), a subset of CD4 T cells that mediates an important role between inflammation and autotolerance (9), partly because of the action of anti-inflammatory cytokines such as interleukin 10 (IL-10) (2). Furthermore, minor expression of the TF *FOXP3* has also been reported in CD8^+^ T cells (10). Another T-box TF family member, Eomesodermin (EOMES), is involved in the polarization of cytotoxic CD4 Th1 cells, also known as CD4 cytotoxic T lymphocytes (CD4 CTLs), which yield the release of cytolytic effector molecules such as granzyme B and perforin and/or the ligation of cell death surface ligands such as FasL (2, 11–13). In addition, it has also been demonstrated that these TFs, particularly T-bet and EOMES, are highly expressed in CD4, CD8β, and γδT cells isolated from the lung of healthy pigs (14). Moreover, they have been related with other functions beyond the regulation of CD4 T-cell differentiation, such as the regulation of cytotoxic CD8^+^ T-cell activity and memory differentiation (1).

Forty years after the appearance of porcine reproductive and respiratory syndrome (PRRS), it is still one of the most economically important diseases in pigs. Because of this, deepening the understanding on the pathogenesis of different PRRS virus (PRRSV) strains continues being a goal for the research community. PRRSV includes two distinct viral species: Betaarterivirus suid 1 (formerly PRRSV-1) and Betaarterivirus suid 2 (formerly PRRSV-2) (15). It has been described that PRRSV suppresses the innate immunity, which yields to an inefficient adaptive immune response in infected pigs (16). The Lena strain, classified as a virulent PRRSV-1 strain, has been shown to exhibit a strong inflammatory immune response in the tracheobronchial lymph node and the lung, together with high severity of lesions (17, 18). Moreover, several studies from our researcher group have shown high rates of cell death in the lung and thymus of pigs infected with virulent PRRSV-1 strains (19–21), which could be partially linked with cytotoxic activity (2, 11–13).

Although the expressions of TFs have been studied in detail in T-cell subsets from different porcine tissues from healthy animals (16, 22, 23), their expressions during PRRSV infection have been poorly studied. Ebner et al. (24) demonstrated a higher frequency of T-bet^+^ CD4^+^ T cells in peripheral blood mononuclear cells (PBMCs) from piglets experimentally infected with PRRSV, together with the overexpression of IFN-γ in *in vitro* stimulated splenocytes. Experimental *in vivo* studies with dendritic cells infected with two different low virulent PRRSV strains showed a significant increase in the expression of the FOXP3 gene, but not for T-bet and GATA3 (25). Moreover, the role of *FOXP3* and Tregs in virulent PRRSV infection is controversial. Comparative *in vivo* experiments have demonstrated that virulent PRRSV-1 strains induced similar or even lower frequencies of Tregs in comparison with low virulent PRRSV-1 strains (26, 27). However, other *in vitro* studies have shown that the virulent PRRSV-2 strain BB0907 induced more CD4^+^CD25^+^Foxp3^+^ Tregs than did a classical PRRSV-2 strain (28).

Considering the role of T-bet, GATA3, FOXP3, and EOMES in T-cell development, differentiation, and memory formation, as well as cytokine production, and the scarcity of studies evaluating their expressions after PRRSV infection, the following work aimed to evaluate the expressions of these TFs in target organs, namely, the lung, tracheobronchial lymph node, and the thymus, and the expressions of different effector cytokines (IFN-γ, TNF-α, and IL-10) and FasL produced by T-cell polarization during the early phase of infection with PRRSV-1 strains of different virulence.

## 2 Materials and Methods

### 2.1 Animals and Experimental Design

The present study is part of a large project carried out to investigate the pathogenesis of PRRSV-1 strains of different virulence. Animals and samples were collected from the experiment published elsewhere (29). Seventy 4-week-old Landrace × Large White piglets, negative against PRRSV (IDEXX PRRS X3 Ab test, IDEXX Laboratories S.L., Barcelona, Spain), *Mycoplasma hyopneumoniae*, and PCV2 [in-house PCR against *M. hyopneumoniae* (30) and PCV2 (31)], were arbitrarily distributed into three different experimental groups at the Centre de Recerca en Sanitat Animal (IRTA-CReSA, Cerdanyola del Vallès, Barcelona, Spain). Briefly, 16 pigs were intranasally inoculated with 2 ml (using MAD Nasal™ Intranasal Mucosal Atomization Device; Teleflex, Alcala de Henares, Madrid, Spain) of porcine alveolar macrophage supernatant diluted in RPMI 1640 medium (Thermo Fisher Scientific, Barcelona, Spain) (control group), 26 pigs with 2 ml of 10^5^ TCID_50_ (50% tissue culture infectious dose) of the low virulent PRRSV-1 3249 strain (subtype 1; 3249 group) (32), and 28 pigs with 10^5^ TCID_50_ of the high virulent PRRSV-1 Lena strain (subtype 3; Lena group) (33). At 1, 3, 6, and 8 days post-infection (dpi), three pigs from the control group and five pigs from each infected group were humanely euthanized. At 13 dpi, four pigs from the control group, six pigs from the 3249, group and eight pigs from the Lena group were euthanized under the same conditions. The experiment was carried out following the guidelines of the European Union (Directive 2010/63/EU) and approved by the Catalan Autonomous Government and by the IRTA Ethics Committee (Project 3647; FUE-2017-00533413). During the necropsies, samples from the lung, tracheobronchial lymph node, and the thymus were collected, immersed in TRIzol™ LS Reagent (Invitrogen, Carlsbad, CA, USA), and frozen at −80°C until processing for RNA extraction. The rest of the tissue sample was fixed in 10% neutral buffered formalin and Bouin’s solution for histopathological and immunohistochemical studies.

### 2.2 Histopathology of the Lung, Tracheobronchial Lymph Node, and Thymus

Four-micrometer hematoxylin and eosin-stained sections from the lung, tracheobronchial lymph node, and the thymus were blindly examined and scored. Evaluation of the severity of lesions in the lung and thymus has been described elsewhere (20, 29). Briefly, interstitial pneumonia and suppurative bronchopneumonia were scored independently, but with the same scale: 0, no microscopic lesions; 1, mild interstitial pneumonia/bronchopneumonia; 2, moderate multifocal interstitial pneumonia/bronchopneumonia; 3, moderate diffuse interstitial pneumonia/bronchopneumonia; and 4, severe interstitial pneumonia/bronchopneumonia. The sum of the interstitial pneumonia and bronchopneumonia scores was considered the final one, being 8 the maximum possible score. The presence of tingible body macrophages in the tracheobronchial lymph node was scored from 0 to 2 as follows: 0, no microscopic changes (<10% of the tissue was affected); 1, mild to moderate microscopic changes (from 10% to 50% of the tissue was affected); and 2, severe microscopic changes (>50% of the tissue was affected). Likewise, the level of depletion on the tissue was scored from 0 to 2. The sum of the presence of tingible body macrophages and the level of lymphoid depletion comprised the final score. The presence of germinal center activation, hemorrhage, inflammatory cells, and mitotic figures was also evaluated. For the thymus, scores were given as follows: 0, no microscopic changes; 1, focal cortical reduction; 2, mild decrease of the ratio cortex/medulla (C/M), multifocal cortical reduction, and presence of tingible body macrophages; 3, moderate decrease of the C/M ratio, multifocal cortical reduction, “starry sky” picture in the cortex, and poor corticomedullary differentiation; and 4, severe decrease of the C/M ratio, disappearance of corticomedullary differentiation with an increase of the stroma, and extensive presence of apoptotic bodies in the cortex.

### 2.3 PCR Analysis

#### 2.3.1 RNA Extraction and cDNA Synthesis

Total RNA was isolated from 100 mg of lung, tracheobronchial lymph node, and thymus homogenized with 2 ml of TRIzol™ LS Reagent using homogenizer 150 (FisherBrand™, Thermo Fisher Scientific) and the NucleoSpin^®^ RNA Virus Column kit (Macherey-Nagel, Düren, Germany) according to the manufacturer’s protocols. In order to remove genomic DNA, a DNase type I Ambion^®^ TURBO-DNA-free™ kit (Life Technologies, Carlsbad, CA, USA) was applied following the manufacturer’s instructions. The concentration and purity of the extracted RNA were determined by spectrophotometry using the Nanodrop 2000 (Thermo Fisher Scientific). One microliter of total RNA was used to generate cDNA using the Script™ cDNA Synthesis Kit (BioRad, Hercules, CA, USA) following the manufacturer’s indications.

#### 2.3.2 PRRSV Viral Load Analysis in the Lung, Tracheobronchial Lymph Node, and Thymus

The LSI™ VetMAX™ PRRSV EU/NA 2.0 kit (Invitrogen) was used to quantify the PRRSV genome according to the manufacturer’s protocol. RT-qPCR reactions were performed in duplicate for each sample in the MyiQ™2 Two-Color Real-Time PCR Detection System (BioRad) for 5 min at 50°C, 10 min at 95°C, followed by 40 cycles of 3 s at 95°C and 30 cycles at 60°C for 30 s. To avoid overestimating the number of viral particles, the results of PRRSV viral load in the lung, tracheobronchial lymph node, and thymus were expressed in quantification cycle (*C*_q_) as previously reported (34).

#### 2.3.3 Relative Quantification of Transcription Factors and Cytokines

Relative quantification of the porcine TFs *T-bet*, *GATA3*, *FOXP3*, and *EOMES*, the cytokines *IFNG*, *TNFA*, and *IL10*, and *FASL* in the lung, tracheobronchial lymph node, and thymus was performed using the comparative *C*_T_ method (also known as the 2^−ΔΔ^ *^C^* ^T^ method). Relative quantification of porcine TFs (*T-bet*, *GATA3*, *FOXP3*, and *EOMES*) and the *IFNG, TNFA, IL10*, and *FASL* results from Lena- and 3249-infected animals are presented as fold change, comparing the value from each infected animal *versus* the average of control animals at each specific time point. The *C*_q_ values of the above-mentioned target genes were normalized to the *C*_q_ values of the reference genes (35). *GeNorm* analysis (qbase+ 2.6.1 software, Biogazelle, Zwijnaarde, Belgium; www.qbaseplus.com) (36) was performed to determine the most stable reference genes from a set of eight reference gene candidates and 10 representative cDNA samples from the lung, tracheobronchial lymph node, and thymus. Three and two reference genes with high stability (average *geNorm M* ≤ 0.5) were established as the optimal reference gene number for lung and tracheobronchial lymph node samples and for thymus samples, respectively. The optimal normalization factor was calculated with the arithmetic mean of ribosomal protein L4 (*RPLA4*), peptidyl-prolyl *cis*–*trans* isomerase A (*PPIA*), and beta-2-microglobulin (*B2M*) in the lung; *RPLA4*, hypoxanthine phosphoribosyl transferase 1 (*HPRT1*), and TATA box-binding protein (*TBP*) in the case of tracheobronchial lymph node; and *RPLA4* and *HPRT1* for the thymus. The sequences of the primers of the porcine reference genes, TFs, cytokines, and FasL are shown in **Table 1**. The primers for *PPIA* and *B2M* were designed using the online *Primer3Plus* tool (www.primer3plus.com) (46). The iTaq™ Universal SYBR Green Supermix kit (BioRad) was used following the manufacturer’s instructions. Reactions were performed in triplicate using 50 ng of cDNA from each sample and 0.5 µM of each primer in the MyiQ™2 Two-Color Real-Time PCR Detection System (BioRad) for 20 s at 95°C for polymerase activation, followed by 40 cycles for denaturation (15 s, 95°C) and annealing/extension (30 s, 60°C). Subsequently, a melting curve analysis was performed (65–95°C) to verify the specificity of amplicons. An inter-run calibrator sample with a known *C*_q_ value was introduced in each plate to guarantee the quality of the retrotranscription and to detect inter-run variations.

**Table 1:** Primer sequences of the porcine reference genes (*RPL4*, *PPIA*, *B2M*, *HPRT1*, and *TBP*) and target genes (*T-bet*, *GATA3*, *FOXP3*, *EOMES*, *IFNG*, *TNFA*, *IL10*, and *FASL*).

*Genes*	*Type*	*Sequences*	*Reference*
*RPL4*	Reference gene	F: 5′-CAAGAGTAACTACAACCTTC-3′	(37)
		R: 5′-GAACTCTACGATGAATCTTC-3′	
*PPIA*	Reference gene	F: 5′-CGCGTCTCCTTCGAGCTGTTT-3′	Self-designed
		R: 5′-GCGTGTGAAGTCACCACCCT-3′	
*B2M*	Reference gene	F: 5′-ACTTTTCACACCGCTCCAGT-3′	Self-designed
		R: 5′-CGGATGGAACCCAGATACAT-3′	
*HPRT1*	Reference gene	F: 5′-GGACTTGAATCATGTTTGTG-3′	(37)
		R: 5′-CAGATGTTTCCAAACTCAAC-3′	
*TBP*	Reference gene	F: 5′-ACGTTCGGTTTAGGTTGCAG-3′	(38)
		R: 5′-GCAGCACAGTACGAGCAACT-3′	
*T-bet*	Target gene	F: 5′-TGCAGTCCCTCCATAAGTACCA-3′	(39)
		R: 5′-GCCTCTGGCTCACCATCATT-3′	
*GATA3*	Target gene	F: 5′-GAGGTCCAGCACAGAAGGCA-3′	(40)
		F: 5′-AAGGGGTCGATTCTGTCCGT-3′	
*FOXP3*	Target gene	F: 5′-CGCATGTTCGCCTTCTTCA-3′	(41)
		R: 5′-AGGCTCAAGTTGTGGCGAAT-3′	
*EOMES*	Target gene	F: 5′-TACGAAACAGGGAAGGCGCA-3′	(42)
		R: 5′-AACGTTGTAGTGGGCAGTAGG-3′	
*IFNG*	Target gene	F: 5′-TGGTAGCTCTGGGAAACTGAATG-3′	(43)
		R: 5′-GGCTTTGCGCTGGATCTG-3′	
*TNFA*	Target gene	F: 5′-CGACTCAGTGCCGAGATCAA-3′	(44)
		R: 5′-CCTGCCCAGATTCAGCAAAG-3′	
*IL10*	Target gene	F: 5′-TGAGAACAGCTGCATCCACTTC-3′	(43)
		R: 5′-TCTGGTCCTTCGTTTGAAAGAAA-3′	
*FASL*	Target gene	F: 5′-CCCATACCCCCAAATCTTCT-3′	(45)
		R: 5′-CTGGACAGGGGAAGACTGAG-3′	

### 2.4 Immunohistochemical Analysis

The immunohistochemical study of IFN-γ, TNF-α, FOXP3, and Fas was performed in the lung, tracheobronchial lymph node, and thymus of animals from control and 3249- and Lena-infected groups euthanized at selected time points according to the RT-qPCR results to identify the main cell subsets implicated in their expression and tissue distribution. In brief, 4-µm tissue sections from each sample were de-waxed and rehydrated in xylene and descending grades of alcohol, respectively, followed by endogenous peroxidase inhibition using 3% H_2_O_2_ in methanol for 30 min in darkness. **Table 2** summarizes the different fixatives, antigen retrieval methods, and dilutions for each antibody. After phosphate-buffered saline (PBS) washes (pH 7.4) and incubation with 100 µl of 2% bovine serum albumin (BSA) for IFN-γ, TNF-α, and Fas and 10% normal goat serum (NGS) for FOXP3, monoclonal primary antibodies were applied and incubated overnight at 4°C in a humidity chamber. For negative controls, the primary antibody was replaced by either an isotype control or by BSA to confirm the lack of nonspecific binding. Thereafter, the slides were washed with PBS and incubated with the corresponding biotinylated secondary antibody diluted in the blocking solution for each case. Labeling was visualized with the NovaRED substrate kit (Vector Elite Laboratories, Burlingame, CA, USA) for IFN-γ, TNF-α, and Fas and with 3,3′-diaminobenzidine chromogen (Dako, Santa Clara, CA, USA) for FOXP3. Finally, the sections were counterstained with Harris hematoxylin, dehydrated, and mounted. In each organ, immunolabeled cells were identified and manually counted by a pathologist in 25 non-overlapping high magnification fields of 0.2 mm^2^ (Olympus BX51, Olympus Iberia SAU, L’Hospitalet de Llobregat, Barcelona, Spain).

**Table 2:** Summary of the immunohistochemical methodology.

Antibody	Type of antibody	Commercial brand	Fixative	Dilution	Antigen retrieval
IFN-γ	pAb	RnD Systems, Minneapolis,MN, USA	Bouin´s solution	1:20	Tween^a^
TNF-α (68B6A3 L1)	mAb	Thermo Fisher, Barcelona, Spain	Bouin’s solution	1:25	Tween^a^
FOXP3 (FJK-16)	mAb	eBioscience™, Barcelona, Spain	10% buffered formalin	1:100	Citrate pH 6^b^
Fas	pAb	Santa Cruz Biotech, Santa Cruz, CA, USA	10% buffered formalin	1:500	Citrate pH 6^c^

pAb, polyclonal antibody; mAb, monoclonal antibody.

a Tween, incubation in Tween-20 diluted in 0.01% phosphate buffered saline for 10 min.

b Citrate pH 6, autoclaved at 121°C for 10 min.

c Citrate pH 6, heat pretreatment in microwave.

### 2.5 Statistical Analyses

Differences between the viral loads in the lung, tracheobronchial lymph node, and thymus and in the relative expressions of TFs (*T-bet*, *GATA3*, *FOXP3*, and *EOMES*), cytokines (*IFNG*, *TNFA*, and *IL10*), and the *FASL* molecule were evaluated for approximate normality of distribution using the D’Agostino and Pearson omnibus normality test, followed by the non-parametric Kruskal–Wallis test for multiple comparisons and the Mann–Whitney non-parametric *U* test for unpaired groups (GraphPad Prism software 7.0, Inc., San Diego, CA, USA). A *p*-value lower than 0.05 was considered statistically significant, indicated with * (*p* ≤ 0.05) and ** (*p* ≤ 0.01). Data are presented as the median ± interquartile range (IQR).

## 3 Results

### 3.1 Virulent PRRSV-1 Lena Strain Induced Severe Lesions in the Lung and Thymus

**Figure 1:**
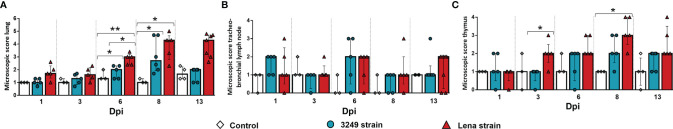
Microscopic findings in the lung, tracheobronchial lymph node, and thymus of each experimental group during the experimental study. Graphs display the microscopic score in the lung **(A)**, tracheobronchial lymph node **(B)**, and thymus **(C)**. *Columns* represent the median ± IQR. Individual values for each animal from the control (*white diamond*), low virulent 3249-infected (*blue circle*), and Lena-infected (*red triangle*) groups are represented. Significant differences between groups are represented (*p ≤ 0.05 and **p ≤ 0.01).

**Figure 2:**
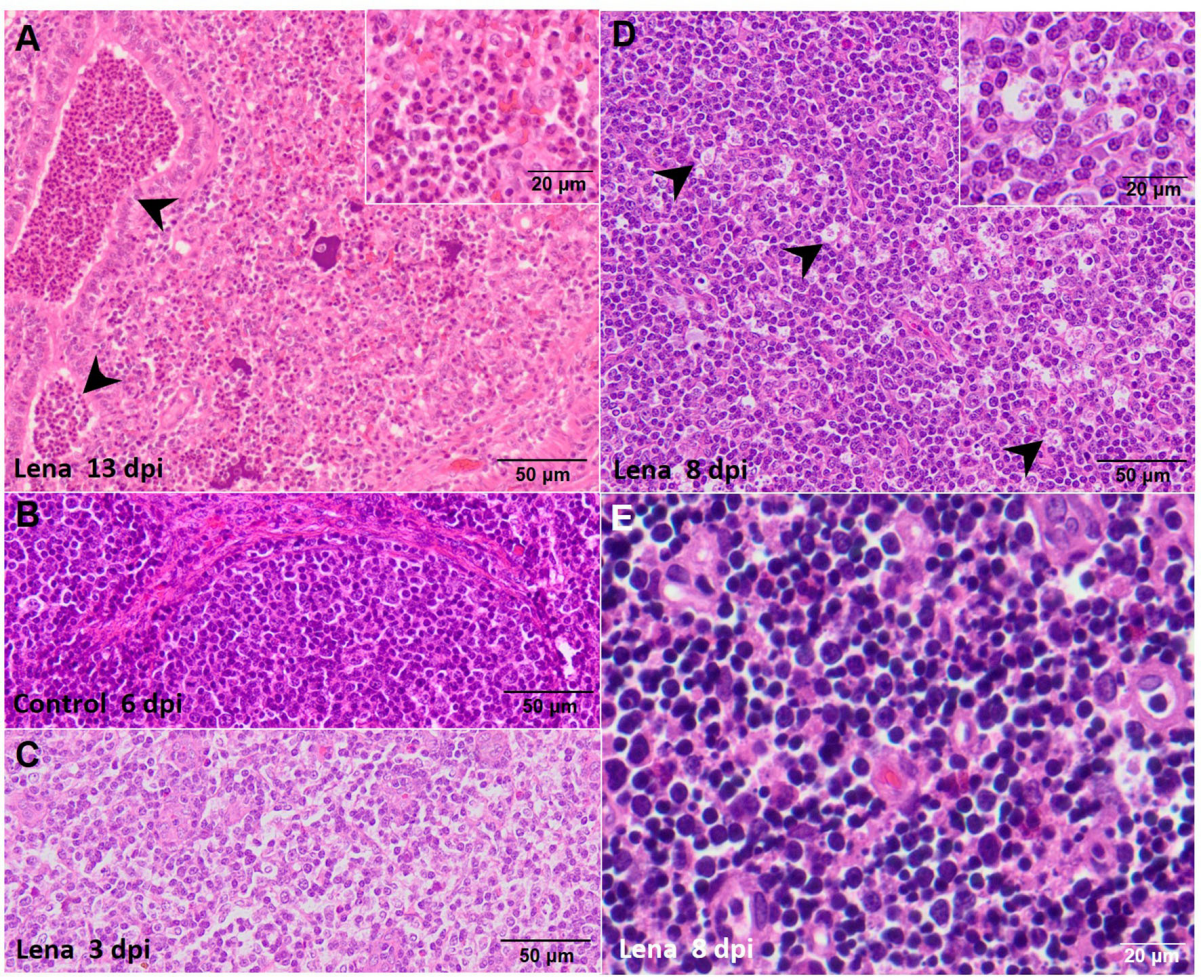
Microscopic pictures of hematoxylin and eosin of representative target organs from infected animals. **(A)** Suppurative bronchopneumonia from a pig infected with the Lena strain and euthanized at 13 days post-infection (dpi) showing a marked intra-alveolar and intra-bronchiolar infiltrate of neutrophils (*black arrowheads*). *Inset* shows the infiltrate of intra-alveolar neutrophils. **(B)** Representative microphotograph of the tracheobronchial lymph node from a pig from the control group euthanized at 6 dpi. **(C)** Lymphoid depletion in the tracheobronchial lymph node from a pig infected with the Lena-strain and euthanized at 3 dpi. **(D)** Numerous tingible body macrophages in the tracheobronchial lymph node (*black arrowheads*) from a pig infected with the Lena strain and euthanized at 8 dpi. *Inset* shows tingible body macrophages. **(E)** Abundant apoptotic bodies in the cortex of the thymus from a pig infected with the Lena strain euthanized at 8 dpi.

Significant differences were observed between the severity of the lesions in the lung from both infected groups in comparison with that in the control group at 6 and 8 dpi (*p* ≤ 0.05 and *p* ≤ 0.01, respectively) (**Figure 1A**), as previously described (29). In brief, these differences consisted of the presence of severe interstitial pneumonia, characterized by a thickening of the alveolar septa together with extensive foci of suppurative bronchopneumonia composed of neutrophils, cell debris, and mucus filling the bronchial, bronchiolar, and alveolar lumen, especially in animals infected with the virulent Lena strain (**Figure 2A**). The microscopic score for the tracheobronchial lymph node was similar between groups, with high individual variability (**Figure 1B**). Minor histological changes were observed when comparing control animals (**Figure 2B**) with both infected groups, except for some infected animals euthanized at 1, 6, 8, and 13 dpi, which presented severe lymphoid depletion (**Figure 2C**) and/or an increase in the number of tingible body macrophages (**Figure 2D**). In the thymus, both infected groups showed a progressive increase in the severity of the lesions; however, the thymus from virulent Lena-infected animals showed significant differences in the microscopic score from 3 dpi onwards in comparison with low virulent 3249-infected animals (*p* ≤ 0.05) (**Figure 1C**). At 8 dpi, the thymus from animals of the virulent Lena group showed severe signs of thymic atrophy characterized by an elevated number of apoptotic bodies in the cortical layer (**Figure 2E**), poor corticomedullar differentiation, and a severe decrease of the C/M ratio with an increase of the stroma [for more details, see (20)].

### 3.2 PRRSV Load Was Higher and Detected Earlier in Tissues From Virulent Lena-Infected Animals

**Figure 3:**
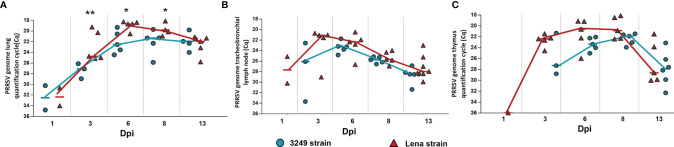
Porcine reproductive and respiratory syndrome virus (PRRSV) viral load in the lung, tracheobronchial lymph node, and thymus of each experimental group during the experimental study. Graphs show PRRSV viral load in the lung **(A)**, tracheobronchial lymph node **(B)**, and thymus **(C)** of infected pigs. Values of PRRSV viral load are represented in quantification cycle (*C*_q_). Individual values for each animal from the low virulent 3249-infected (*blue circle*) and Lena-infected (*red triangle*) groups are represented. Significant differences between groups are represented (*p ≤ 0.05 and **p ≤ 0.01).

PRRSV was not detected in any studied organ from control animals along the study. Similar viral kinetics was observed in the lung, tracheobronchial lymph node, and thymus from Lena- and 3249-infected animals, but the increase occurred earlier and was higher in those infected with the virulent Lena strain throughout the study (**Figure 3**). PRRSV was detected in the lung as early as 1 dpi in two out of five animals from both infected groups (**Figure 3A**). Significant differences between Lena- and 3249-infected pigs (*p* ≤ 0.01 at 3 dpi; *p* ≤ 0.05 at 6 and 8 dpi) were observed in the PRRSV load, reaching the maximum at 6 dpi in virulent Lena-infected animals (*C*_q_ = 18.68, IQR = 1.27) and at 8 dpi in those infected with the low virulent 3249 strain (*C*_q_ = 22.07, IQR = 3.89) and reducing these differences at 13 dpi (Lena group: *C*_q_ = 21.95, IQR = 3.23; 3249 group: *C*_q_ = 22.74, IQR = 2.71) (**Figure 3A**). In the tracheobronchial lymph node, on the contrary, the virus was detected as early as 1 dpi, but only in two out of five animals from the virulent Lena group (*C*_q_ = 27.6, IQR = 5.06) (**Figure 3B**). Whereas all animals from this group were positive (*C*_q_ = 21.07, IQR = 4.41) at 3 dpi, only three out of five animals from the low virulent 3249 group were positive at this date. At 6 dpi, the 3249 group peaked (*C*_q_ = 23.1, IQR = 1.85) and the Lena group showed similar value to that detected at 3 dpi (*C*_q_ = 22.35, IQR = 3.51). Then, a progressive decrease was observed in both infected groups until the end of the study (*C*_q_ = 27.96, IQR = 3.14 for the Lena group and *C*_q_ = 28.29, IQR = 1.39 for the 3249 group at 13 dpi). No statistical differences were found between both infected groups regarding the PRRSV load in the tracheobronchial lymph node (**Figure 3B**). At 1 dpi, PRRSV was detected in the thymus from only one virulent Lena-infected pig (*C*_q_ = 36.01), followed by a strong increase at 3 dpi, where the PRRSV viral load was detected in the thymus from all virulent Lena-infected animals and only in three out of five pigs infected with low virulent 3249 (**Figure 3C**). Both infected groups showed similar viral kinetics, peaking at 8 dpi (Lena-infected animals: *C*_q_ = 20.87, IQR = 4.05; 3249-infected animals: *C*_q_ = 22.57, IQR = 2.05) and dropping at 13 dpi (Lena-infected animals: *C*_q_ = 28.61, IQR = 7.26; 3249-infected animals: *C*_q_ = 28.86, IQR = 5.34). No significant differences in PRRSV viral load were detected in the thymus of both infected groups (**Figure 3C**).

### 3.3 *T-bet*, *IFNG*, and *TNFA* Genes Were Overexpressed in Tissues, Mainly Thymus From the Virulent Lena-Infected Group Since the First Week Post-Infection

**Figure 4:**
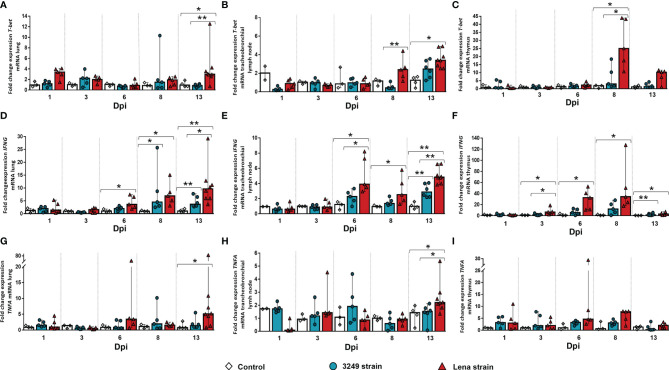
Relative mRNA expressions of *T-bet*, *IFNG*, and *TNFA* in target organs. Graphs show fold change expressions of *T-bet*, *IFNG*, and *TNFA* in the lung (**A**, **D**, **G**, respectively), tracheobronchial lymph node (**B**, **E**, **H**, respectively), and thymus (**C**, **F**, **I**, respectively). Relative quantification was performed with the *C*_T_ method (also known as the 2^−ΔΔ^ *^C^* ^T^ method). *Columns* represent the median ± IQR. Individual values for each animal from the control (*white diamond*), 3249-infected (*blue circle*), and Lena-infected (*red triangle*) groups are represented. Significant differences between groups are represented (*p ≤ 0.05 and **p ≤ 0.01).

A marked peak in *T-bet* expression in the lung of virulent Lena-infected animals (fold change = 3.01, IQR = 1.42) was observed at 13 dpi with significant differences compared with the low virulent 3249-infected group (*p* ≤ 0.01) and the control group (*p* ≤ 0.05) (**Figure 4A**). *T-bet* was significantly overexpressed from 8 dpi onwards in the tracheobronchial lymph node of virulent Lena-infected animals in comparison with low virulent 3249-infected animals (*p* ≤ 0.01) (**Figure 4A**) (Lena-infected animals: fold change = 2.32, IQR = 2.09; 3249-infected animals: fold change = 0.42, IQR = 0.47). An increase in the expression of *T-bet* at 13 dpi was also observed in the tracheobronchial lymph node of piglets infected with the 3249 and Lena strains (3249-infected group: fold change = 2.45, IQR = 1.93; Lena-infected group = 3.4, IQR = 2.96), although only statistically significant in Lena-infected animals (*p* ≤ 0.05) (**Figure 4B**). In the thymus, a striking peak of *T-bet* expression was detected at 8 dpi in the Lena-infected group compared with the 3249-infected group (Lena-infected animals: fold change = 25.01, IQR = 29.49; 3249-infected animals: fold change = 3.03, IQR = 12.50; *p* ≤ 0.05), followed by a drastic drop at 13 dpi (fold change = 10.27, IQR = 7.05) (**Figure 4C**).

Regarding the expression level of *IFNG*, a progressive increase in the lung of virulent Lena-infected animals from 6 dpi (fold change = 3.59, IQR = 4.74) to 13 dpi (fold change = 9.63, IQR = 6.41) was observed (*p* ≤ 0.05 at 6, 8, and 13 dpi) (**Figure 4D**). This raise was also noticed in low virulent 3249-infected pigs at 8 dpi (*p* ≤ 0.05), mildly decreasing at the end of the study (fold change = 3.64, IQR = 3.83; *p* ≤ 0.01) (**Figure 4D**). An increase in the gene expression of this cytokine was observed in the tracheobronchial lymph nodes from both infected groups from 6 dpi (Lena-infected animals: fold change = 3.89, IQR = 4.53; 3249-infected animals: fold change = 2.27, IQR = 1.70) until the end of the study (fold change = 4.82, IQR = 2.01 for Lena-infected animals and fold change = 2.86, IQR = 1.82 for 3249-infected animals at 13 dpi), but was always higher in virulent Lena- than that in low virulent 3249-infected pigs (**Figure 4E**). Significant differences between the virulent Lena and control groups were found at 6, 8, and 13 dpi and between the low virulent 3249 and control groups at 13 dpi (*p* ≤ 0.01 and *p* ≤ 0.05) (**Figure 4E**). The expression of *IFNG* in the thymus from virulent Lena-infected piglets showed a progressive increase from 3 dpi (fold change = 6.43, IQR = 10.17, *p* ≤ 0.05) until 8 dpi (fold change = 34.22, IQR = 67.86, *p* ≤ 0.05), undergoing a significant drop at 13 dpi (fold change = 2.87, IQR = 5.20, *p* ≤ 0.05) (**Figure 4F**). In the thymus from low virulent 3249-infected group, a similar kinetics was observed, but with a lower intensity than that in virulent Lena-infected group, with significant differences observed only at 13 dpi with respect to the control group (fold change = 1.42, IQR = 3.13, *p* ≤ 0.01) (**Figure 4F**).

*TNFA* in the lung of virulent Lena-infected animals showed two peaks, one at 6 dpi (fold change = 3.34, IQR = 12.49) and the other at 13 dpi (fold change = 5.10, IQR = 7.83, *p* ≤ 0.05) (**Figure 4G**). No significant changes were found in the *TNFA* kinetics of the lung from low virulent 3249-infected animals (**Figure 4G**). *TNFA* in the tracheobronchial lymph node showed basal expression in both infected groups compared to the control group, except for the last day of study when an increased expression was observed only in virulent Lena-infected piglets (fold change = 2.20, IQR = 1.03, *p* ≤ 0.05) (**Figure 4H**). In the thymus, the main changes were observed in the expression of *TNFA* at 8 dpi (fold change = 7.77, IQR = 4.63), decreasing onwards in the virulent Lena-infected group, but with wide individual variability and no statistically significant changes (**Figure 4I**). A basal expression of *TNFA* was noted in the thymus from low virulent 3249-infected animals along the whole study (**Figure 4I**).

**Figure 5:**
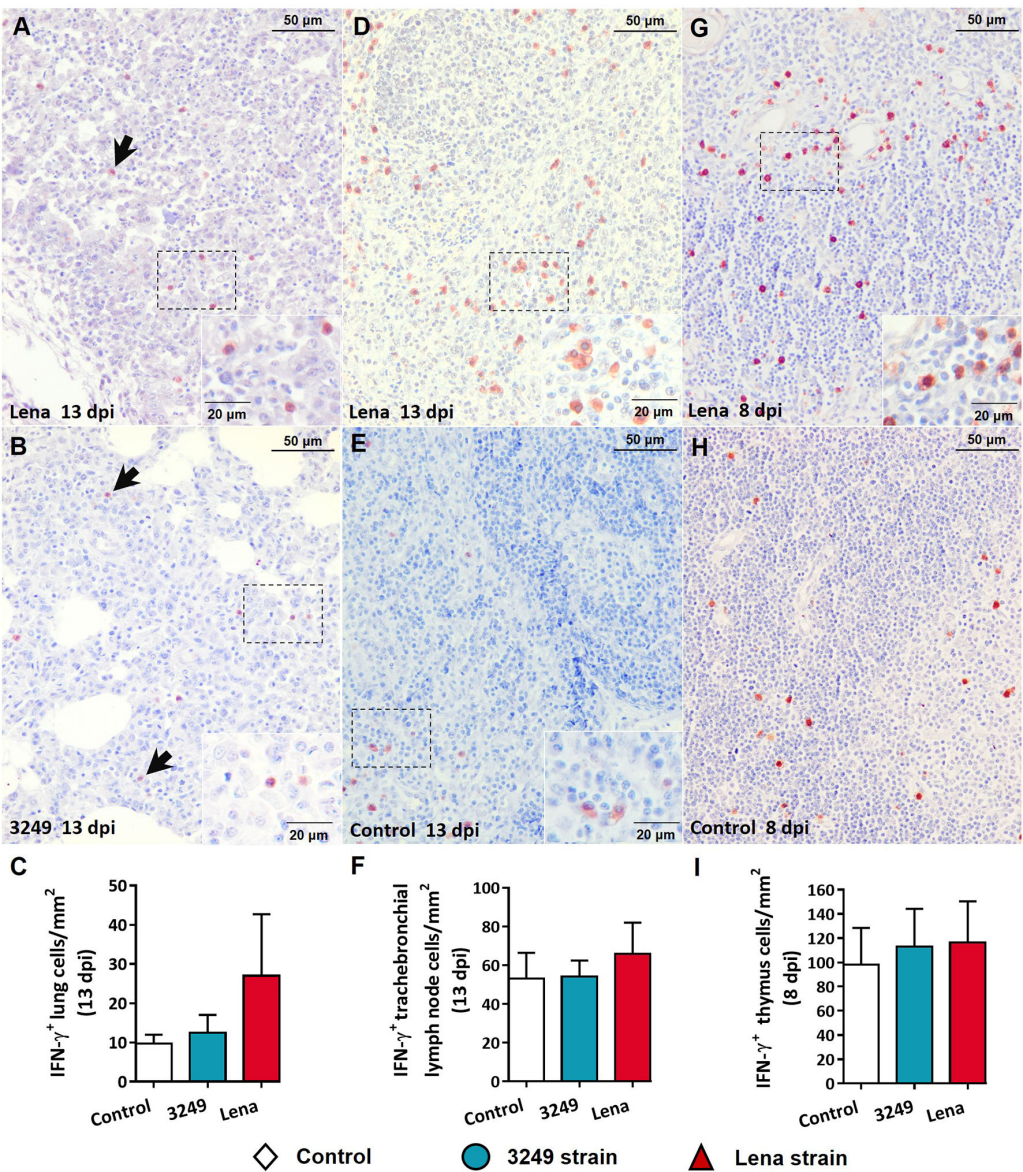
Immunohistochemical expression and counting of IFN-γ in target organs. **(A, B)** Photomicrographs against IFN-γ of the lung from Lena-infected **(A)** and 3249-infected **(B)** piglets euthanized at 13 days post-infection (dpi) showing pulmonary alveolar macrophages and interstitial macrophages (*black arrows*) in foci of interstitial pneumonia and suppurative bronchopneumonia. *Insets* show pulmonary alveolar macrophages. **(C)** Graph showing the number of IFN-γ^+^ cells in the lung. **(D, E)** Photomicrographs against IFN-γ of the tracheobronchial lymph node from a piglet infected with the Lena strain **(D)** and from a control piglet **(E)** euthanized at 13 dpi. *Insets* show lymphocytes and macrophages from the paracortex and medulla expressing IFN-γ. **(F)** Graph showing the number of IFN-γ^+^ cells in the tracheobronchial lymph node. **(G, H)** Photomicrographs against IFN-γ of thymus from a piglet infected with the Lena strain **(G)** and from a control piglet **(H)** euthanized at 8 dpi. *Inset* shows thymocytes and macrophages from the medulla expressing IFN-γ. **(I)** Graph showing the number of IFN-γ^+^ cells in the tracheobronchial lymph node. *Columns* represents the median with range from the control group (*white*), low virulent 3249-infected (*blue*), and Lena-infected (*red*) groups.

Immunohistochemical labeling of IFN-γ revealed a greater number of immunostained cells in tissues from virulent Lena-infected piglets in comparison with low virulent 3249-infected piglets and control piglets (**Figure 5**). Pulmonary alveolar macrophages and interstitial macrophages were the main subsets immunolabeled against IFN-γ in the lung (**Figures 5A, B** **)**, particularly in virulent Lena-infected animals (**Figure 5A**). In the tracheobronchial lymph node, the IFN-γ protein was mainly detected in the cytoplasm of lymphocytes located in the medulla and paracortex (**Figures 5D, E** **)**. In the thymus, the expression of IFN-γ was observed mainly in thymocytes and macrophage-like cells of the thymic medulla from virulent Lena-infected piglets (**Figure 5G**), and to a lesser extent in low virulent 3249-infected and control piglets (**Figure 5H**).

**Figure 6:**
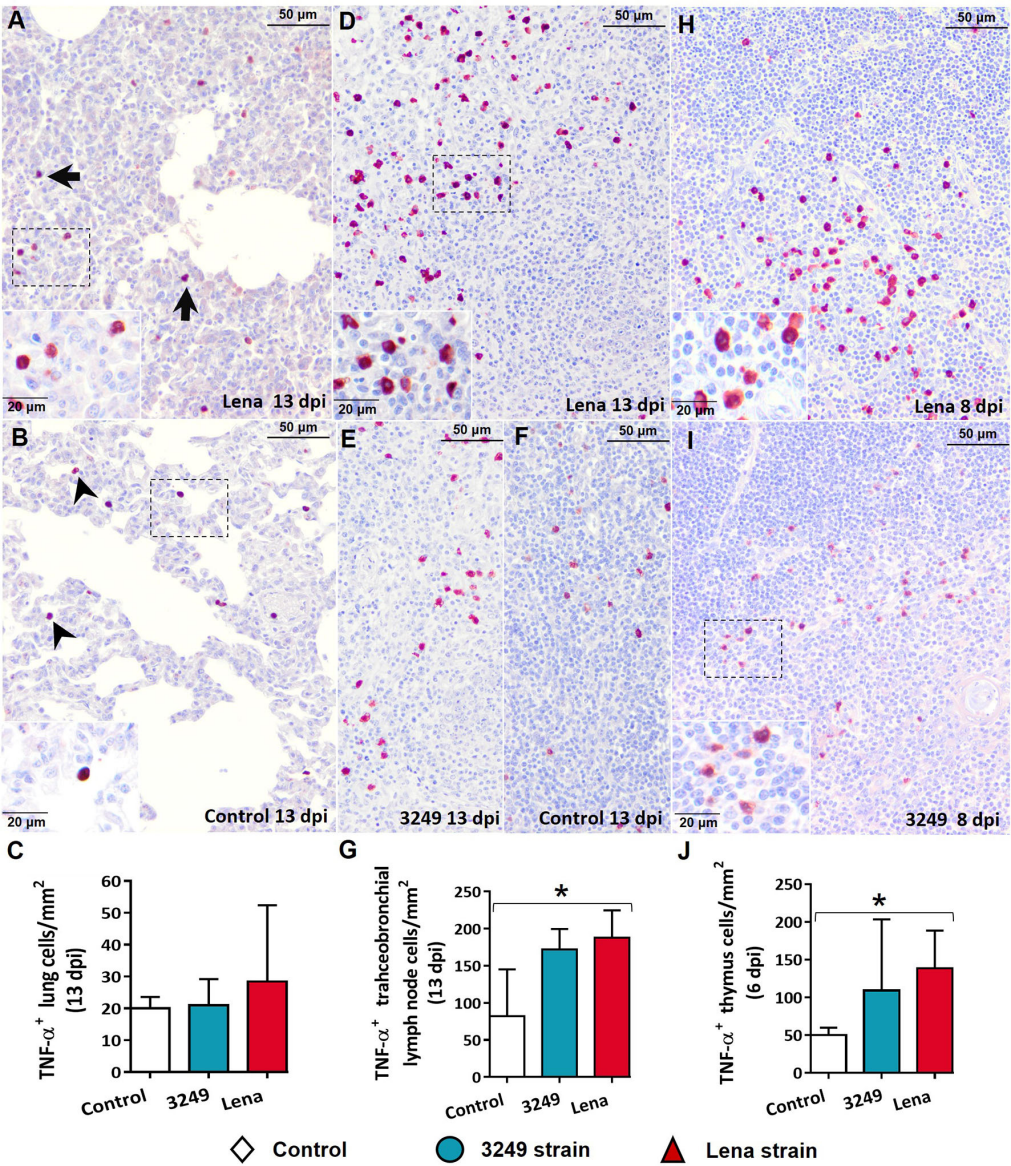
Immunohistochemical expression and counting of TNF-α in target organs. **(A, B)** Photomicrographs against TNF-α of the lung from a Lena-infected pig **(A)** and from a control pig **(B)** euthanized at 13 days post-infection (dpi) showing interstitial macrophages (*black arrows*) and pulmonary alveolar macrophages (*black arrowheads*) and in foci of suppurative bronchopneumonia and interstitial pneumonia and. *Insets* show interstitial macrophages (*top*) and pulmonary alveolar macrophages (*bottom*). **(C)** Graph showing the number of TNF-α^+^ cells in the lung. **(D–F)** Photomicrographs against TNF-α of the tracheobronchial lymph node from a pig infected with the Lena strain **(D)**, from a piglet infected with 3249 **(E)**, and from a control piglet **(F)** euthanized at 13 dpi. Inset *shows* lymphocytes and macrophages from the medulla expressing TNF-α. **(G)** Graph showing the number of TNF-α^+^ cells in the tracheobronchial lymph node. **(H, I)** Photomicrographs against TNF-α of the thymus from Lena-infected **(H)** and 3249-infected **(I)** piglets euthanized at 8 dpi. *Inset* shows thymocytes and macrophages from the corticomedullary border and medulla expressing TNF-α. **(J)** Graph showing the number of TNF-α^+^ cells in the thymus. *Columns* represents the median with range from the control group (*white*), low virulent 3249-infected (*blue*), and Lena-infected (*red*) groups. Significant differences between groups are represented (*p ≤ 0.05).

Immunolabeling against TNF-α was mainly detected in tissues from virulent Lena-infected piglets in comparison with low virulent 3249-infected piglets and control animals (**Figure 6**). Interstitial macrophages from areas of interstitial pneumonia of the infected animals (**Figure 6A**) and pulmonary alveolar macrophages from the lung of control animals (**Figure 6B**) were the main populations involved in TNF-α expression. In the tracheobronchial lymph node, labeling revealed TNF-α^+^ lymphocytes in the medulla of tracheobronchial lymph nodes from both infected groups (**Figures 6D, E** **)**, but mainly in those from virulent Lena-infected animals (**Figure 6D**). In the thymus, the expression of TNF-α was detected in the corticomedullary border and medulla from both infected groups (**Figures 6H, I** **)** but mainly in those infected with the virulent Lena strain (**Figure 6H**). Significant differences in the number of TNF-α^+^ cells were detected between the control and Lena-infected group in the tracheobronchial lymph node and thymus (*p* ≤ 0.05).

### 3.4 No Significant Changes Were Observed in *GATA3* Expression in the Different Tissues From Virulent Lena- and Low Virulent 3249-Infected Piglets

**Figure 7:**
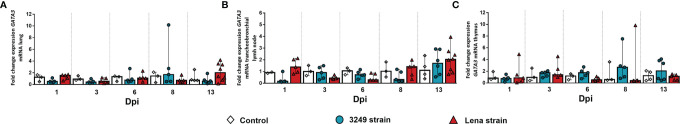
Relative mRNA expression of *GATA3* in target organs. **(A–C)** Graphs showing fold change expression of *GATA3* in the lung **(A)**, tracheobronchial lymph node **(B)**, and thymus **(C)**. Relative quantification was performed using the *C*_T_ method (also known as the 2^−ΔΔ^ *^C^* ^T^ method). *Columns* represent the median ± IQR. Individual values for each animal from the control (*white diamond*), 3249-infected (*blue circle*), and Lena-infected (*red triangle*) groups are represented.

The expression of *GATA3* in the studied organs from infected groups was low, and no differences were found with respect to the control group (**Figure 7**). A marked individual variability was observed between animals, particularly in the thymus.

### 3.5 *FOXP3* Transcription Factor Was Overexpressed in Tissues From Both Infected Groups at 13 dpi

**Figure 8:**
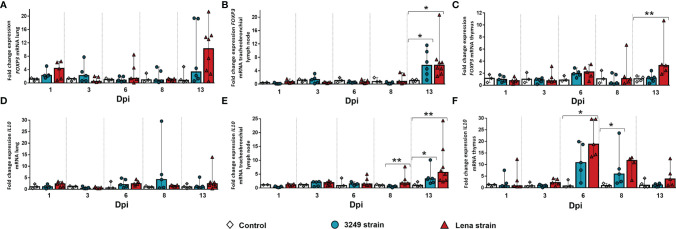
Relative mRNA expressions of *FOXP3* and *IL10* in target organs. Graphs show fold change expressions of *FOXP3* and *IL10* in the lung (**A**, **D**, respectively), tracheobronchial lymph node (**B**, **E**, respectively), and thymus (**C**, **F**, respectively). Relative quantification was performed using the *C*_T_ method (also known as the 2^−ΔΔ^ *^C^* ^T^ method). *Columns* represent the median ± IQR. Individual values for each animal from the control (*white diamond*), 3249-infected (*blue circle*), and Lena-infected (*red triangle*) groups are represented. Significant differences between groups are represented (*p ≤ 0.05 and **p ≤ 0.01).

An overexpression in the *FOXP3* gene was detected in the lung and tracheobronchial lymph nodes from both infected groups at 13 dpi (**Figures 8A, B** **)**. However, statistically significant differences between infected groups and the control group were only detected in the tracheobronchial lymph node (virulent Lena-infected animals: fold change = 5.63, IQR = 3.67; low virulent 3249-infected animals: fold change = 5.58, IQR = 7.60; *p* ≤ 0.05) (**Figure 8B**). In thymus, in contrast, the increase in the expression of *FOXP3* was only detected in virulent Lena-infected animals at 13 dpi (fold change = 3.28, IQR = 4.91, *p* ≤ 0.01) (**Figure 8C**).

Regarding the expression of the *IL10* gene in the lung, no significant differences were observed, except for a peak at 8 dpi in the lung from low virulent 3249-infected pigs, but with large individual variability (fold change = 4.22, IQR = 17.30) (**Figure 8D**). A high increase was observed at 13 dpi in the tracheobronchial lymph nodes from both virulent Lena- and low virulent-infected piglets, but to a lesser extent in the latter (Lena-infected group: fold change = 5.62, IQR = 9.57; 3249-infected group: fold change = 3.26, IQR = 3.34; *p* ≤ 0.01 and ≤ 0.05, respectively) (**Figure 8E**). The peak in the expression of *IL10* was observed earlier (at 6 dpi) in the thymus from both infected groups, being higher in the thymus from virulent Lena-infected animals (Lena-infected animals: fold change = 18.78, IQR = 15.71; 3249-infected animals: fold change = 10.86, IQR = 14.45) and decreasing onwards (**Figure 8F**).

**Figure 9:**
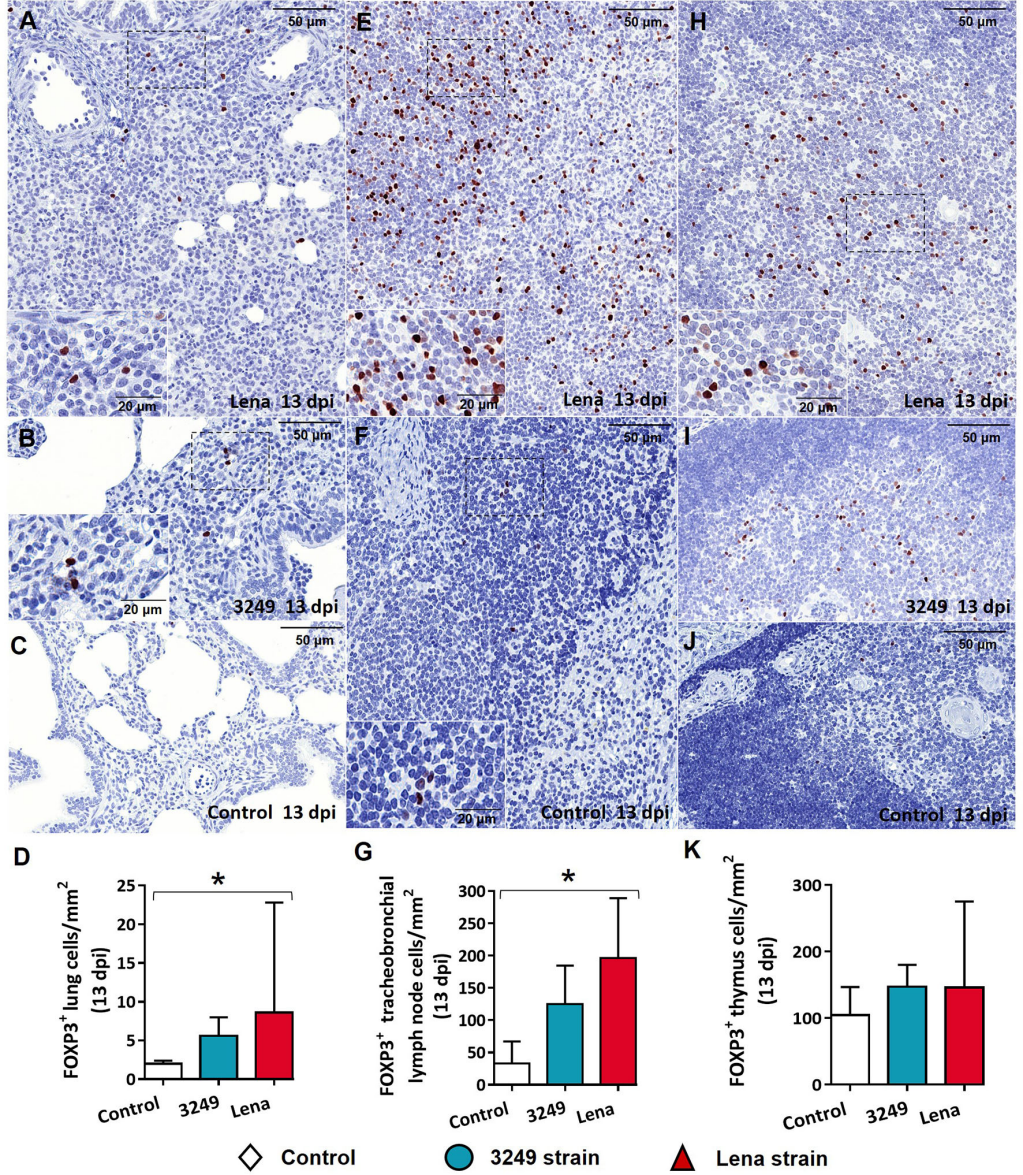
Immunohistochemical expression and counting of FOXP3 target organs. **(A–C)** Photomicrographs against FOXP3 of the lung from Lena-infected **(A)**, 3249-infected **(B)**, and control **(C)** piglets euthanized at 13 days post-infection (dpi). *Insets* show lymphocytes expressing FOXP3 in foci of interstitial pneumonia. **(D)** Graph showing the number of FOXP3^+^ cells in the lung. **(E, F)** Photomicrographs against FOXP3 of the tracheobronchial lymph node from a piglet infected with the Lena strain **(E)** and from a control piglet **(F)** euthanized at 13 dpi. *Insets* show lymphocytes from the paracortex and medulla expressing FOXP3. **(G)** Graph showing the number of FOXP3^+^ cells in the tracheobronchial lymph node. **(H–J)** Photomicrographs against FOXP3 of the thymus from Lena-infected **(H)**, 3249-infected **(I)**, and control **(J)** piglets euthanized at 13 dpi. *Inset* shows lymphocytes from the medulla expressing FOXP3. **(K)** Graph showing the number of FOXP3^+^ cells in the thymus. *Columns* represents the median with range from the control group (*white*), low virulent 3249-infected (*blue*), and Lena-infected (*red*) groups. Significant differences between groups are represented (*p ≤ 0.05).

The immunolabeling of FOXP3 in the lung was observed in the nuclei of lymphocytes mainly located in areas of interstitial pneumonia in both Lena- and 3249-infected groups (**Figures 9A, B** **)**. In the tracheobronchial lymph node, FOXP3 was evident in the nuclei of lymphocytes from the cortex and paracortex (**Figures 9E, F** **)**. Animals from both infected groups evidenced a higher number of FOXP3^+^ cells in comparison with animals from the control group (**Figure 9G**). Significant differences were found between the control and Lena-infected groups in the FOXP3^+^ cell counts in the lung and tracheobronchial lymph node (*p* ≤ 0.05) (**Figures 9D, G** **)**. In the case of the thymus, thymocytes from the medulla of both groups of infected animals (**Figures 9H, I** **)** showed a higher expression of FOXP3 than did the control animals (**Figures 9J, K**). Lung from control group showed low expression of FOXP3^+^ cells (**Figure 9C**). In general, FOXP3^+^ cells were more numerous in target organs from virulent Lena-infected pigs than from low virulent 3249-infected animals.

### 3.6 A High Expression of *EOMES* and *FASL* Genes Were Detected at 13 dpi in Target Organs From Virulent Lena-Infected Animals

**Figure 10:**
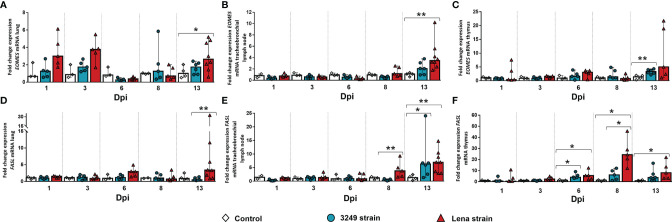
Relative mRNA expressions of *EOMES* and *FASL* in target organs. Graphs show fold change expressions of *EOMES* and *FASL* in the lung (**A**, **D**, respectively), tracheobronchial lymph node (**B**, **E**, respectively), and thymus (**C**, **F**, respectively). Relative quantification was performed with the *C*_T_ method (also known as the 2^−ΔΔ^ *^C^* ^T^ method). *Columns* represent the median ± IQR. Individual values for each animal from the control (*white diamond*), 3249-infected (*blue circle*), and Lena-infected (*red triangle*) groups are represented. Significant differences between groups are represented (*p ≤ 0.05 and **p ≤ 0.01).

The expression of the *EOMES* gene in the lung from virulent Lena-infected animals increased at 1, 3, and 13 dpi in comparison with the control group (fold change = 3.00, IQR = 3.27; fold change = 3.76, IQR = 1.94; and fold change = 2.65, IQR = 3.14, respectively), but only at 13 dpi were there significant differences (*p* ≤ 0.01) due to the wide individual variability along the study (**Figure 10A**). In the case of the tracheobronchial lymph node, *EOMES* was observed at 13 dpi in the tracheobronchial lymph nodes from both infected groups, but greater in those from piglets infected with the virulent Lena strain (Lena-infected animals: fold change = 3.47, IQR = 2.56; 3249-infected animals: fold change = 2.06, IQR = 2.29 for; *p* ≤ 0.01) (**Figure 10B**). *EOMES* was lightly expressed in the thymus during the whole experiment, with an increase in both infected groups only at the end of the study (3249-infected pigs: fold change = 3.31, IQR = 0.73; Lena-infected pigs: fold change = 5.00, IQR = 18.59; *p* ≤ 0.01) (**Figure 10C**).

Regarding *FASL*, a statistically significant increase in its expression was observed in the lung from virulent Lena-infected animals at the end of the study (fold change = 3.38, IQR = 8.94, *p* ≤ 0.05), whereas it presented baseline levels in the lung from low virulent 3249-infected animals along the study (**Figure 10D**). *FASL* was significantly overexpressed in the tracheobronchial lymph node from virulent Lena-infected piglets from 8 dpi onwards (fold change = 3.80, IQR = 5.41 at 8 dpi; fold change = 6.96, IQR = 6.98 at 13 dpi; *p* ≤ 0.01) (**Figure 10E**). At 13 dpi, *FASL* was also overexpressed in the lymph node from pigs infected with the low virulent 3249 strain (fold change = 6.31, IQR = 8.79, *p* ≤ 0.01) compared with the control group (**Figure 10E**). In the thymus from virulent Lena-infected animals, the expression of *FASL* displayed a curve peaking at 8 dpi (fold change = 24.38, IQR = 20.86) and dropping at 13 dpi (fold change = 8.09, IQR = 14.48, *p* ≤ 0.05) (**Figure 10F**). In the thymus from low virulent 3249-infected animals, only a mild increase of *FASL* was detected from 6 dpi (fold change = 4.15, IQR = 3.98, *p* ≤ 0.05), which was maintained until the end of the study (fold change = 3.72, IQR = 8.25) (**Figure 10F**).

**Figure 11:**
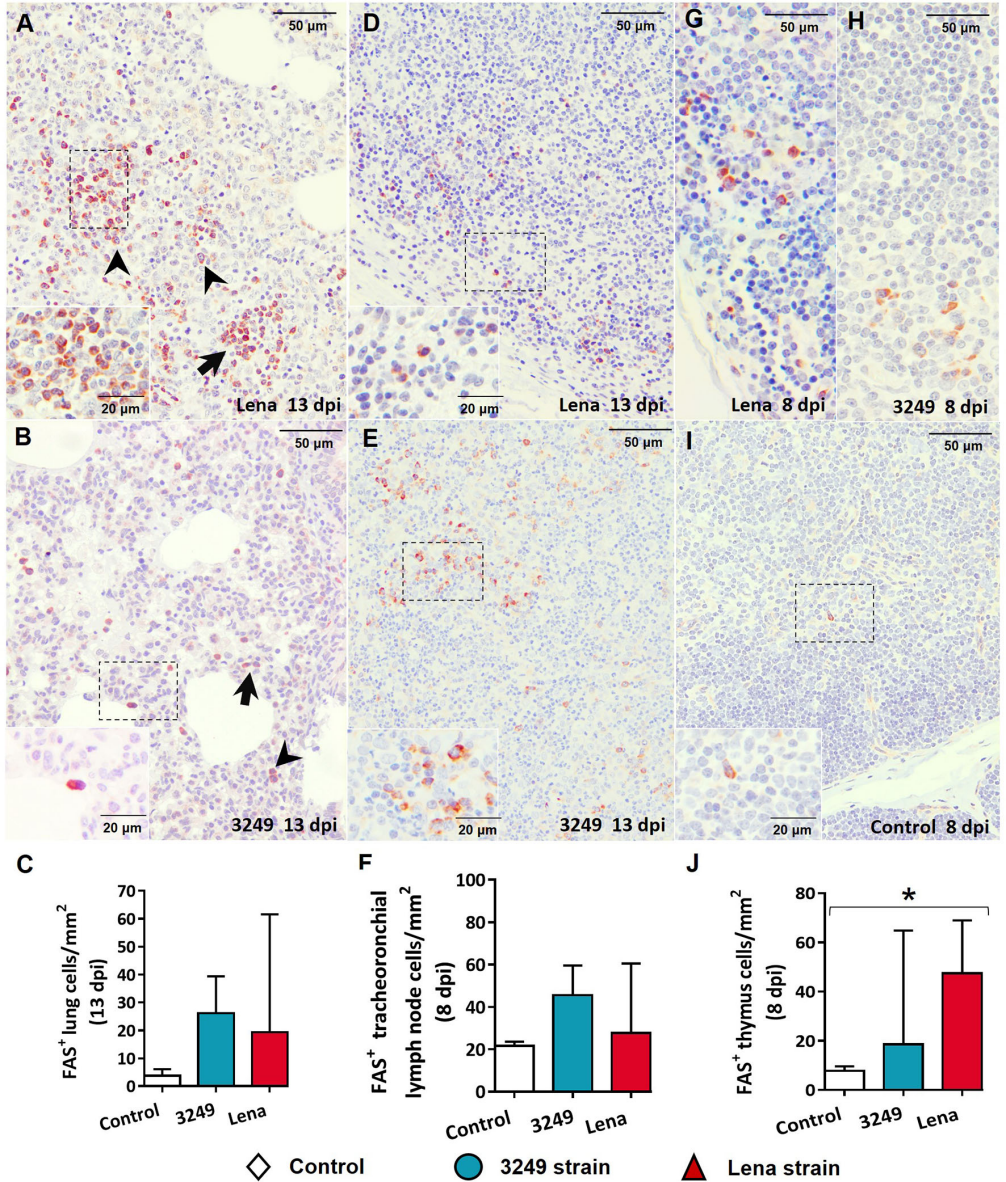
Immunohistochemical expression and counting of Fas in target organs. **(A, B)** Photomicrographs against Fas of the lung from Lena-infected **(A)** and 3249-infected **(B)** animals euthanized at 13 days post-infection (dpi) showing interstitial macrophages (*black arrowheads*) and lymphocytes (*black arrows*) in foci of interstitial pneumonia. *Insets* show lymphocytes (*top*) and pulmonary alveolar macrophages (*bottom*). **(C)** Graph showing the number of Fas^+^ cells in the lung. **(D, E)** Photomicrographs against Fas of the tracheobronchial lymph node from Lena-infected **(D)** and 3249-infected **(E)** piglets euthanized at 13 dpi. *Insets* show lymphocytes from the medulla and paracortex expressing Fas. **(F)** Graph showing the number of Fas^+^ cells in the tracheobronchial lymph node. **(G–I)** Photomicrographs against Fas of the thymus from Lena-infected **(G)**, 3249-infected **(H)**, and control **(I)** piglets euthanized at 8 dpi. *Insets* show lymphocytes from the medulla and thymic cortex expressing Fas. **(J)** Graph showing the number of Fas^+^ cells in the thymus. *Columns* represents the median with range from the control group (*white*), low virulent 3249-infected (*blue*), and Lena-infected (*red*) groups. Significant differences between groups are represented (*p ≤ 0.05).

In the lung, Fas^+^ cells consisted of lymphocytes and interstitial and pulmonary alveolar macrophages from areas of interstitial pneumonia (**Figures 11A, B** **)**, mainly in animals from the virulent Lena-infected group (**Figure 11A**). These cells were also observed in the lung from low virulent 3249-infected animals, but to a lesser extent (**Figure 11B**). Lung from control group showed low expression of Fas+ cells (**Figure 11C**). Fas labeling was detected in the cytoplasm of lymphocytes and macrophage-like cells in the medulla and paracortex of the tracheobronchial lymph nodes from both infected groups (**Figures 11D–F** **)**. In the thymus, Fas expression was mainly evidenced in thymocytes of the thymic medulla from both infected groups (**Figures 11G, H** **)** in comparison with the control group (**Figures 11I, J**), but also in the cortex from virulent Lena-infected animals (**Figure 11G**).

## 4 Discussion

The crosstalk between T cells and antigen-presenting cells (APCs) plays a key role in the establishment of adaptive immunity in lymph nodes (47). TFs regulate the development of different immune cell subsets by activating or repressing genes that are critical to cellular identity (3). Moreover, TFs control the genes that are involved in cell-type-specific proliferative and migratory properties, metabolic features, and effector functions (3). In this study, we evaluated the expressions of different TFs (*T-bet*, *GATA3*, *FOXP3*, and *EOMES*) through RT-qPCR and the associated cytokine profiles during infection with PRRSV-1 strains of different virulence in target organs.

The expression of the *T-bet* gene was upregulated in the lung, tracheobronchial lymph node, and thymus of virulent Lena-infected animals from 8 dpi onwards in comparison with the low virulent 3249 and control groups. This TF has been pointed out as the key regulator of CD4^+^ Th1 cells due to its role not only of genetically reprogramming CD4^+^ Th1 cells but also suppressing CD4^+^ Th2 cells (7). A low expression of *T-bet* has been noticed in the thymus from control and low virulent infected animals in our study, as was previously detected in γδT cells of the thymus from healthy pigs in other studies (23). However, a marked upregulation of the *T-bet* transcripts was observed in the thymus from the Lena-infected group at 8 dpi, which could be playing a role in the context of virulent PRRSV infection in this organ. This thymic upregulation of *T-bet* could be related with the migration of these cells from the periphery to the thymus, mainly from regional lymph nodes as priming organs. Another explanation could be related to the development of innate T cells, among which are γδT cells, mucosal-associated invariant T cells, and NK T cells (48, 49), as already speculated for other viral diseases (50), although further studies would be required to confirm this hypothesis in the context of PRRSV infection.

*T-bet* expression is induced by antigen receptor-derived signals and inflammatory cytokines, such as IL-12, IL-27, and IFN-γ (3, 7). Interestingly, in our study, a peak of *IFNG* expression was observed before that of *T-bet* expression (6-8 dpi), which was more marked in the target organs from Lena-infected animals. A previous study revealed a high proportion of CD4^+^ T cells co-expressing *T-bet* and IFN-γ in comparison with uninfected animals, which was suggestive of a Th1 polarization induced by the low virulent CReSA 3267 PRRSV-1 strain (24). Beyond this commitment, T-bet was also expressed by other immune cell types, including CD8^+^ T cells, γδT cells, DCs, or NK cells, being required for the proper functioning of these cells and acting as a bridge between innate and adaptive immune responses (14, 23, 48, 51). Thus, *T-bet* promotes the effector differentiation of CD8 T cells, playing a minor role during memory formation (3). A high frequency of *T-bet* in CD8^+^ T cells has been reported in lung from healthy pigs, whereas the percentages were rather low in the mediastinal lymph node (14). In our study, enhanced expressions of *T-bet* in the lung and tracheobronchial lymph node from the Lena-infected group in comparison with the low virulent and control groups were detected in our study. The greater expressions of *T-bet* in the tracheobronchial lymph node and lung at the end of this study would suggest the traveling of activated Th1 and effector CD8^+^ T cells from the former (8 dpi) to the latter (13 dpi) in order to face the infection with the virulent Lena strain. Interestingly, CD8 T cells were the primary source of IFN-γ production in the lung after vaccination with the virulent PRRSV-21-7-4 strain (52).

Our results suggest that the virulent Lena strain induces a stronger and earlier Th1 and effector CD8^+^ T-cell polarization than does the low virulent 3249 strain in target organs. In agreement with this result, Bordet et al. (53) reported a higher *in vitro* activation of conventional type 1 dendritic cells (cDC1) induced by the virulent Lena strain in comparison with low virulent strains, which suggested a Th1 response also accompanied by high expressions of T-bet, IFN-γ, and IL-12. The activation of the Th1 and effector CD8 phenotypes by the upregulation of the T-bet gene is also supported in our study by the overexpression of the cytokine *TNFA* in the tracheobronchial lymph node and lung of virulent Lena-infected animals at the end of the study. This pro-inflammatory cytokine has been associated with apoptosis and other cell death phenomena in the context of PRRSV infection, specifically during infection with the virulent Lena strain, not only in the lung but also in the thymus (20, 21), helping to explain the severity of the lesions associated with this strain. Whereas IFN-γ^+^ and TNF-α^+^ cells were mainly located in lymphocytes from the medullar area in the tracheobronchial lymph node and thymus, pointing out lymphoid lineage (expressing the T-bet gene) as the major source of IFN-γ and TNF-α in these organs, the expressions of these cytokines in the lung were mostly evidenced in interstitial and pulmonary alveolar macrophages, mainly associated with local inflammatory response.

GATA3 has been shown to be essential for both T-cell development and Th2 cell fate (8, 54–56). However, during the whole study, no differences in the expression of *GATA3* were observed in the thymus, neither in tracheobronchial lymph node nor in the lung, from control and both infected groups. A previous report was able to evidence an increase in the frequency of *GATA3* CD4 T cells following infection with *Trichuris suis*, but, in agreement with our result, was not demonstrated after PRRSV infection (24).

A high expression of the *FOXP3* gene, the main modulator of CD4^+^ Tregs (9), was also observed in the tracheobronchial lymph node and lung at the end of the study. Two types of FOXP3^+^ Tregs could affect the immunopathogenesis during viral infections (57). Natural Tregs are generated in the thymus and help to prevent autoimmunity, whereas activated or induced Tregs are responsible for limiting tissue damage and inflammation in peripheral tissues and are associated with innate and adaptive immune responses (57–59). Increased frequencies of CD4^+^ Tregs have been observed in a huge number of studies on human and animal viral infections and have been associated with the establishment of persistent infection (2, 60). Porcine *in vitro* studies have demonstrated that low virulent PRRSV strains are able to induce Tregs (25, 60, 61), which has been associated with IL-10 production in subsequent studies (60, 62, 63). The expression of IL-10 could favor viral persistence and secondary complications in the lungs from infected piglets (62). However, *in vivo* studies with virulent PRRSV-1 strains have released controversial results regarding the induction of Tregs during the early phase of infection. Whereas no evident increase in the frequency of Tregs was observed in pigs infected with virulent PR40/2014 and SU1 PRRSV-1 strains in comparison with low virulent strains (26, 27), our research group already described an increase in FOXP3 expression in the lung from Lena-infected pigs at 2 weeks post-infection (18). In the present study, the same kinetics in the expression of the *FOXP3* gene in the tracheobronchial lymph node was observed for *IL10*. These Tregs could be the source of IL-10, which could mean an attempt to modulate the expressions of the other cytokines, such as IFN-γ and TNF-α, as mentioned above, observed in this organ. In the thymus from low virulent 3249-infected animals, the expression of the *FOXP3* gene was constant along the whole study. However, an induction of natural Tregs could be speculated in the thymus from virulent Lena-infected animals due to the higher expression of the *FOXP3* gene in this group in comparison with the control and low virulent 3249-infected groups at the end of the study (13 dpi). Additionally, *FOXP3* has been demonstrated to be expressed in CD8^+^ T cells from the mesenteric lymph nodes and thymus of healthy pigs (22); however, these CD8^+^ *FOXP3* ^+^ cells have been demonstrated to be low IFN-γ inducers in other studies (64).

The *EOMES* gene, the main TF of CD4 CTLs, was overexpressed in target organs from virulent Lena-infected animals at the end of the study. This effector CD4^+^ T-cell subset is developed under inflammation and infection, being induced during antiviral response to promote virus clearance through cytotoxicity and cytokine-dependent mechanisms (2, 13). However, depending on their maturation stage, CD4 CTLs could also be regulated by T-bet (13). Particularly, EOMES induces CD4^+^ T cytotoxicity by activating the perforin and FasL pathways (2, 11–13). Curiously, in our study, a peak of the *FASL* gene was demonstrated in the lung and tracheobronchial lymph node from virulent Lena-infected piglets parallel to the expression of *EOMES*, which was also shown through immunohistochemical expression of Fas. The interaction of Fas/FasL mediates the cleavage of caspase-8, a mediator of extrinsic apoptotic pathway (65), whose activation has been previously described in the lung and thymus from animals infected with the virulent Lena strain (20, 21). Moreover, EOMES, together with T-bet, could also be co-expressed in naïve and effector CD8^+^ T cells, which promotes IFN-γ, perforin, and granzyme B expressions (3, 66). In the thymus from virulent Lena-infected animals, a high expression of the *EOMES* gene was detected at the end of the study (13 dpi), but with wide individual variability and no significant differences with respect to the other groups. Nevertheless, earlier expression of the *FASL* gene was detected in the thymus from both infected groups (6 dpi), with a peak of expression at 8 dpi in the thymus of virulent Lena-infected piglets coinciding with the severity of lesions in this organ. A similar kinetics to this one was also observed in the expression of the Fas protein in the thymus from virulent Lena-infected animals in a parallel study from our research group (20). The expression of the *FASL* gene observed in our study could be associated with an indirect induction by pro-apoptotic cytokines, such as TNF-α, a hypothesis supported by the enhancement of the expression of the *TNFA* gene observed in our study.

In the present study, we have observed high expressions of *T-bet*, *EOMES*, and *FOXP3*, which are suggestive of a polarization toward Th1 cells and Tregs, but also toward CD4^+^ CTLs, effector CD8^+^ T cells, and γδT cells particularly in response against the virulent PRRSV-1 Lena strain. Although our findings suggest an activation of some T-cell subsets, it would be of interest to study the kinetics of expression of such TFs during the late stages of PRRSV infection to better understand the immunobiology of this disease.

## Data Availability Statement

The raw data supporting the conclusions of this article will be made available by the authors, without undue reservation.

## Ethics Statement

The animal study was reviewed and approved by the IRTA Ethics Committee and by the Catalan Autonomous Government (Project 3647; FUE-2017-00533413) and carried out following the European Union guidelines (Directive 2010/63/EU).

## Author Contributions

IR-G, JG-L, and LC conceived, designed, and performed the project. FP, IR-G, and JG-L helped in the animal experiments and sample collection. IR-T, JS-C, FL-M, and IB conducted the laboratory experiments and analyzed the data. IR-T wrote the manuscript. JG-L and IR-G reviewed the manuscript. LC, FP, and JG-L supervised the study and contributed to reagents/materials/analysis tools. All authors contributed to the article and approved the submitted version.

## Funding

This work was supported by the Spanish Ministry of Economy and Competitiveness (#AGL2016-76111-R and PID2019-109718GB-I00).

## Conflict of Interest

The authors declare that the research was conducted in the absence of any commercial or financial relationships that could be construed as a potential conflict of interest.

## Publisher’s Note

All claims expressed in this article are solely those of the authors and do not necessarily represent those of their affiliated organizations, or those of the publisher, the editors and the reviewers. Any product that may be evaluated in this article, or claim that may be made by its manufacturer, is not guaranteed or endorsed by the publisher.
